# Reactions to Problematic Internet Use Among Adolescents: Inappropriate Physical and Mental Health Perspectives

**DOI:** 10.3389/fpsyg.2020.01782

**Published:** 2020-07-31

**Authors:** Cheng-Min Chao, Kai-Yun Kao, Tai-Kuei Yu

**Affiliations:** ^1^Department of Business Administration, National Taichung University of Science and Technology, Taichung, Taiwan; ^2^Department of Business Administration, Hwa Hsia University of Technology, New Taipei, Taiwan; ^3^Department of Business Administration, National Quemoy University, Jinning, Taiwan

**Keywords:** community bond, problematic Internet use, inappropriate physical and mental health, mental health, structure equation model (SEM)

## Abstract

Although numerous studies have examined the factors influencing problematic Internet use (PIU), few studies have investigated the interactions between inappropriate physical and mental health (e.g., cyberbullying, Internet pornography, and Internet fraud) as factors facilitating PIU and examined the moderating effect of community bond. Thus, this study analyzed the moderating role of community bond in the relationship between cyberbullying, Internet pornography, Internet fraud, and PIU. Using a cross-sectional survey, adolescents were surveyed through self-report questionnaires. A total of 5,211 responses were received from participant students at 60 senior high schools in Taiwan. Statistical analyses were performed using structural equation modeling. The results indicated that cyberbullying, Internet pornography, Internet fraud, and community bond have significant positive effects on PIU. Community bond has a significant moderating effect in the relationship between cyberbullying, Internet fraud, and the PIU of adolescents. Parental Internet attitude and behavior were found to significantly moderate the relationship between inappropriate physical and mental health, community bond, and PIU. The results suggest that public health and education policies should focus more on adolescents who require additional assistance. Furthermore, school policies could be more informed in regard to relevant psychosocial variables and patterns of Internet use. Finally, this study may serve as a reference for parents, schools, and government education authorities.

## Introduction

Since the 1990s, the increasing prevalence of the Internet has made this technological tool central to everyday life and changed how people interact and communicate (Błachnio and Przepiorka, 2016; Kaya and Bicen, 2016). Simultaneously, the Internet has become an action situated in the digital context (Musetti and Corsano, 2018) for socializing, academic research, acquiring new information, entertainment, and healthcare concerns. Consequently, research in this area has grown rapidly, particularly over the last decade. Appropriate Internet use, notably through facilitating social communication and access to information and knowledge, may be beneficial for the development and mental health of individuals and causes positive outcomes for the large majority of users. However, the multifunctional nature of the Internet also causes negative behaviors. If overused, the Internet may hinder development, damage mental health and social functioning, and even cause addiction.

The different terms for and definitions of Internet addiction share the common aspect of negativity toward the individual. However, problematic Internet use (PIU) specifically means that an individual uses the Internet excessively resulting in negative outcomes, and clinical judgment is required to determine whether an individual is addicted. Moreover, cognitive–behavioral researchers often use PIU to describe the middle of the problem severity continuum and stress the benign and moderate qualities of its related negative outcomes (e.g., truancy). By contrast, addiction researchers consider Internet addiction to be at the upper end of the continuum, involving serious negative life outcomes (e.g., marriage failure, dropping out of school, and losing a job) (Tokunaga, 2014; Pontes et al., 2016). In recent years, few studies have explored the relationship between cyberbullying, Internet pornography, and Internet fraud and PIU. Past research indicates that cyberbullying, Internet pornography, and Internet fraud have become negative activities on the Internet and may have an impact on mental health. Therefore, this study proposed a novel notion: the impact of inappropriate physical and mental health on PIU. However, despite many researchers acknowledging PIU as a critical problem among adolescents (Spada, 2014; Dunbar et al., 2017; Lai and Kwan, 2017; Wartberg et al., 2017; Musetti et al., 2020), controversy remains regarding the boundary between PIU and Internet addiction. For the sake of consistency in this study, we employed the more conservative term problematic Internet use instead of Internet addiction.

Adolescents, for whom the Internet is an indispensable part of their daily life, are the most significant PIU-risk group (Lam, 2015; Öztürk and Özmen, 2016; Faghani et al., 2020; Sela et al., 2020). According to several epidemiological studies (Moreno et al., 2013; Mihara et al., 2016; Lai and Kwan, 2017; Wartberg et al., 2017), the prevalence estimates of PIU vary widely. Globally, the reported prevalence estimate of adolescents with PIU has ranged from 0.8% in Italy to 26.7% in Hong Kong (Kuss et al., 2014; Mihara et al., 2016; Wartberg et al., 2017). Although the number of relevant studies is limited, the prevalence estimate of PIU appears to be higher in Asian countries than in others (Kuss et al., 2014; Wartberg et al., 2017). Mihara et al. (2016) conducted a nationwide survey in adolescent participants who were randomly selected from schools in Japan. A total of 100,050 students were randomly selected from junior and senior high schools. The estimated prevalence rate of PIU was 7.9%, and PIU was more prevalent in female than male students (9.8 versus 6.2%) and differed among countries. A similar pattern has also been identified in Taiwan, one of Asia’s leading economies. According to a representative national sample, approximately 10.6–15.3% of university students are addicted to the Internet (Lin et al., 2011; Wu et al., 2015). In conclusion, the estimated prevalence of PIU is relatively high, especially in Asia. PIU has become a serious public health concern that is recognized worldwide, and greater attention must be paid to adolescents’ PIU (Lai and Kwan, 2017; Wartberg et al., 2017). To address the potential harm of PIU to adolescents’ mental health, parents, teachers, and numerous other parties require additional information.

In the past decade, information and communication technologies (ICTs) have drastically changed the manner in which individuals and social groups communicate, interact, and exchange information. The negative nature of these online activities can damage and distort adolescents’ mental development (Kor et al., 2014; Arora, 2016; Lazuras et al., 2017). Many previous studies have indicated that cyberbullying, Internet pornography, and Internet fraud may negatively affect multiple aspects of adolescents’ mental health and behavioral problems (Kor et al., 2014; AlBuhairan et al., 2017; Allen et al., 2017; Lazuras et al., 2017; Moreno-Fernández et al., 2017; Savage and Tokunaga, 2017).

In the last decade, PIU has received attention from researchers, schools, and parents. Family functioning, parent–child relationships, family relationships, and parental socioeconomic backgrounds are known to be connected with the risky behaviors of adolescents and have been a focus of many studies (Pontes et al., 2016; Lai and Kwan, 2017; Wartberg et al., 2017; Musetti et al., 2020; Sela et al., 2020). Numerous studies have examined the effects of these four factors on individuals’ behavior. Musetti et al. (2020) research indicates that adolescents who feel lonely in their relationships with parents and emotionally detached from them manifest more PIU. They argued that adolescents’ lack of perceived parental support may exposes the adolescents to unpleasant emotions, thereby enhancing compulsive–impulsive Internet use as a maladaptive coping strategy (Musetti et al., 2020). Yen et al. (2009) suggested that a lower level of parental monitoring is statistically associated with PIU in adolescents. Nevertheless, no existing study has attempted to investigate the possible effects of parental Internet behavior, parental attitude toward Internet use, and Internet cognition on adolescent PIU. Therefore, further investigation is required into the effects on PIU of cyberbullying, Internet pornography use, and Internet fraud, as well as the interaction effect on PIU of community bond, cyberbullying, Internet pornography use, Internet fraud by parental Internet behavior, parental attitude toward Internet use, and Internet cognitive status.

To summarize, PIU has become a significant problem worldwide, especially among adolescents. The effect of adolescents’ inappropriate physical and mental health, such as cyberbullying, Internet pornography use, and Internet fraud, on PIU is a critical topic to be discussed. The current study attempted to construct a theoretical model that can predict and explain the effects of adolescent PIU and also empirically tested the model. Researchers have reported that PIU has become a critical public health problem worldwide, especially adolescent PIU (Lai and Kwan, 2017; Wartberg et al., 2017); however, no empirical study has explored the effects of inappropriate physical and mental health (e.g., cyberbullying, Internet pornography use, and Internet fraud) on PIU. To fill this gap, the current study developed a measurement scale for use with senior high school students (including vocational high school students) in Taiwan. The questionnaire survey method was adopted to determine how inappropriate physical and mental health affect adolescent PIU. Therefore, the purposes of the present study were as follows: (1) to investigate one primary antecedent (cyberbullying, Internet pornography use, and Internet fraud) for PIU; (2) to examine whether cyberbullying, Internet pornography use, Internet fraud, and community bonds moderate or predict PIU; and (3) to examine the effects of parental Internet attitudes, such as parental use of the Internet, parental restrictions on Internet use, and parents’ evaluation of the Internet’s influence on their children, on PIU. In this context, the research questions of this study were defined as follows: (1) Can inappropriate physical and mental health, such as cyberbullying, Internet pornography use, and Internet fraud, significantly predict PIU? (2) How does the community bond moderate the effects of cyberbullying, Internet pornography use, and Internet fraud on PIU? (3) How do parental Internet attitudes and behaviors, such as parental use of the Internet, parental restrictions on Internet use, and parents’ evaluation of the Internet’s influence on their children, affect the PIU in adolescents? The results are expected to enhance scholarly understanding of PIU and adolescents’ inappropriate physical and mental health, such as cyberbullying, Internet pornography use, and Internet fraud to provide references for parents, schools, and government education authorities and to elicit the effects of psychology education and related problems.

## Literature Review

### Problematic Internet Use

The concept of Internet addiction was first introduced by Young (1998) in a study for the American Psychological Association. Young (2004) defined Internet addiction as an impulse control disorder that does not require an intoxicant, which makes it a behavioral addiction similar to gambling addiction but different from alcoholism. Internet addiction is now a well-known term but lacks standardized diagnosis. Musetti et al. (2016) point out that the Internet addiction disorder (IAD) proposed by Young (1996) was formulated before the development of Internet as intended in current society. In addition, it was not clearly defined, and the structure is too broad and general to make a definite diagnosis (Musetti et al., 2016). Therefore, many researchers have used numerous terms and definitions to depict the status of addicts, including virtual addiction, compulsive computer use, Internet dependence, pathological Internet use, and problematic Internet use (PIU) (Spada, 2014; Wong et al., 2015; Dunbar et al., 2017; Lai and Kwan, 2017; Faghani et al., 2020).

Shapira et al. (2000) described PIU as when an individual cannot control their urge and increasing tension to access the Internet, which finally causes feelings of pain or dismay and negative life consequences. Davis (2001) indicated that PIU refers to the problematic use of the Internet by an individual for a specific purpose (e.g., online gaming, online sex, and online gambling). PIU has been defined as a lack of the strength to limit Internet use despite severe negative outcomes in daily life (Tam and Walter, 2013; Spada, 2014). Douglas et al. (2008) defined PIU as an individual’s inability to control their impulse to overuse the Internet, which in turn leads to feelings of distress and functional impairment of daily activities. Lai and Kwan (2017) defined PIU as excessive use of the Internet that causes disturbances or harm to the individual. Accordingly, in this research, PIU is defined as an unhealthy (non-clinical) use of the Internet that negatively influences an individual’s daily life.

### Inappropriate Physical and Mental Health

#### Cyberbullying

Cyberbullying is a growing phenomenon that potentially affects the daily lives of numerous adolescents worldwide and raised concern among the public. The prevalence of cyberbullying victimization among adolescents has been estimated to range between 8 and 40% (Savage and Tokunaga, 2017). Consequently, cyberbullying among adolescents has gained considerable global attention (AlBuhairan et al., 2017; Lazuras et al., 2017; Savage and Tokunaga, 2017; Camerini et al., 2020). Several studies have indicated that cyberbullying may negatively affect multiple aspects of adolescents’ mental health (e.g., causing depression, social anxiety, suicide, and low self-esteem) and behavioral problems (e.g., deterioration of relationships between family members and a decrease in grades) (Dredge et al., 2014; AlBuhairan et al., 2017; Lazuras et al., 2017; Savage and Tokunaga, 2017; Camerini et al., 2020). A survey by Ditch the Label (2016) discovered that people who have been bullied are almost twice as likely to bully others; 44% of young people who have been bullied experience depression; and 33% of those who have been bullied have suicidal thoughts. Based on the above discussion, the rise in cyberbullying has been facilitated by the ease of producing online content, the ability to post messages anonymously, fast and widespread dissemination, and the absence of the pressures associated with face-to-face communication. The evolution of this new form of bullying deserves the attention of researchers because it causes great harm to bullied adolescents and affects their personality development.

#### Internet Pornography

The Internet provides a wealth of information and possibilities for adolescents; however, like in any other areas of life, potential dangers and risks also exist. The easy access to and abundance of pornographic content online may increase anxiety about the harmful influence of Internet pornography on adolescents (D’Orlando, 2011; Hald et al., 2013; Kor et al., 2014; Allen et al., 2017); thus, the multitude of pornographic websites and their potentially negative consequences have attracted public concern. Consequently, research on Internet pornography has become much more popular. Internet pornography is different from other forms of pornography because of its accessibility and privacy, as well as its often being available at no cost (Kor et al., 2014). Advances in ICT have not only made pornography more accessible but may have also accelerated the societal acceptance of pornography use (D’Orlando, 2011; Kor et al., 2014). Children and adolescents are the most frequent users of ICT in a family. However, mental problems have been associated with frequent use of pornography (Hald et al., 2013). Therefore, the exposure of children and adolescents to Internet pornography is especially worrying because it can cause negative psychological problems in the development of such individuals and stimulate higher acceptance of sexual permissiveness, as well as lead to sexual activity at an early age, sexual compulsivity, pornography addiction, and engagement in risky sexual behavior (Hald et al., 2013; Kor et al., 2014; Allen et al., 2017). Although Internet pornography has gained much attention from scholars, little actual research has been conducted. A large proportion of existing research has empirically examined the effects of numerous determinants on students who access pornography and who have problematic pornography use (Allen et al., 2017; Brown et al., 2017). Hald et al. (2013) asserted that mental problems have also been linked to frequent access to pornography. To our knowledge, no empirical study has directly examined the relationship between Internet pornography and PIU. Therefore, this study aims to understand the effect of Internet pornography on PIU in adolescents.

#### Internet Fraud

Information technology has advanced rapidly in recent decades, along with the rapid growth in the number of Internet users. Although the Internet provides numerous benefits and has changed society positively, it has also created a space in which criminals can operate, created new avenues for criminal pursuits, and facilitated new forms of victimization (Vahdati and Yasini, 2015; Arora, 2016). Furthermore, although fraudulent commercial transactions have long been a problem, advances in the Internet have transformed their dynamics and provided numerous alternative methods for conducting criminal activity online (Aleem and Antwi-Boasiako, 2011). Therefore, Internet users may become victims of Internet fraud, sometimes without even being aware of it. Internet fraud differs from traditional fraud in that the Internet is the instrument with which the crime is committed and can be used to commit more complex variations of traditional crimes. Internet fraud is one of the forms of computer crime that is increasing in prevalence most rapidly. Internet fraud refers to fraud schemes that take advantage of one or more feature of the Internet to commit a crime (Vahdati and Yasini, 2015). These features can be websites, email, message boards, or chat rooms. As a relatively new form of crime, Internet fraud affects the daily lives of numerous people. Internet fraud can occur in multiple forms (Aleem and Antwi-Boasiako, 2011; Vahdati and Yasini, 2015; Arora, 2016; Moreno-Fernández et al., 2017), such as advance fee fraud schemes, credit or debit card fraud, spoofing and phishing, spam, and Internet auction fraud. Among these, Internet auction fraud is the most ubiquitous type of Internet fraud (Aleem and Antwi-Boasiako, 2011; Vahdati and Yasini, 2015; Arora, 2016). Although Internet fraud has become a matter of global interest and importance and gained much attention from scholars, little research has been conducted, and literature is sparse. A large proportion of existing research that has explored the effects of Internet fraud has often only discussed financial losses and the factors affecting Internet fraud (Aleem and Antwi-Boasiako, 2011; Vahdati and Yasini, 2015; Arora, 2016; Moreno-Fernández et al., 2017). To our knowledge, no empirical study has directly examined the relationship between Internet fraud and PIU. Accordingly, in this study, we investigated the influence of Internet fraud on PIU among adolescents.

### Community Bond

Online social networking sites (SNSs) have become one of the most popular activities for people accessing the Internet and form a part of youths’ identity. Studies (Barker, 2012; Ceglarek and Ward, 2016) have asserted that adolescents use the Internet and SNSs to explore, shape, and grow their social identities through social interactions and self-presentation. The rapid growth of SNSs has been followed by the development of many different social networking platforms, such as online communities. Online communities are a relatively new way of building relationships between remote individuals. Many researchers have acknowledged the potential of online communities and the significant roles they play in different areas (Fiedler and Sarstedt, 2014; Ivaturi and Chua, 2019; Tsai and Hung, 2019). Online communities and SNSs enable people to meet friends; meet new people; exchange ideas, videos, and pictures; and even engage in commercial economic activities. When individuals are described as members of a community, it makes them feel good, and they feel a strong sense of attachment to the entire community. Teo et al. (2003) point out that experienced members who have devoted more time to build relationships and have become the most engaged users because of their interests and knowledge are the major participants in these communities.

From the perspective of interpersonal relationship research, when adolescents participate in online communities, the direct contact between adolescents and other members leads to strong interpersonal bonds, which reinforce group cohesion (Buchan et al., 2006; Fiedler and Sarstedt, 2014). Therefore, the participation of adolescents in online communities contributes to the development of interpersonal relationships, establishment of long-term relationships with new members, and establishment of a collective identity through mutual sharing. When the identity of adolescents’ online community is established, not only more new information from online communities are obtained but also group cohesion is reinforced. Based on the above discussion, this study argued that when adolescents obtain cyberbullying, Internet pornography, and Internet fraud-related information in the online community, the community bond can positively moderate the impact of cyberbullying and Internet pornography on adolescents’ PIU. In addition, the online community provide many new methods of Internet fraud. Therefore, this study argued that community bonds can negatively moderate the impact of Internet fraud on adolescents’ PIU.

## Materials and Methods

### Research Model

**FIGURE 1:**
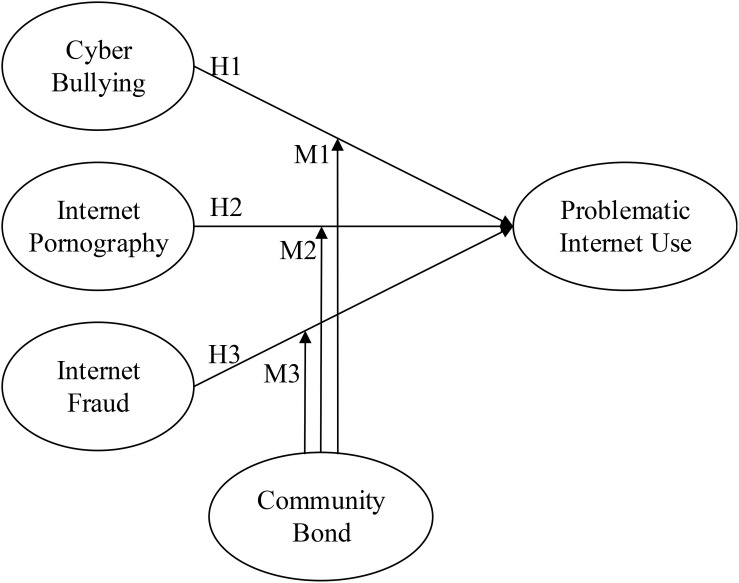
Research framework.

The present study examined whether cyberbullying, Internet pornography use, Internet fraud, and community bond moderate or predict PIU. The hypothesized model were constructed using (1) the exogenous variables (cyberbullying, Internet pornography use, and Internet fraud), (2) the endogenous variable (PIU), and (3) the moderator variable (community bond). The hypotheses are numbered and illustrated in the proposed path model in Figure 1.

### Research Hypotheses

The following hypotheses were proposed:

H1: Cyberbullying has a significant effect on PIU.
H2: Internet pornography use has a significant effect on PIU.
H3: Internet fraud has a significant effect on PIU.
M1: The relationship between cyberbullying and PIU is moderated by level of community bond.
M2: The relationship between Internet pornography use and PIU is moderated by level of community bond.
M3: The relationship between Internet fraud and PIU is moderated by level of community bond.

### Measurement Tool Development and Validation

The questionnaire comprised previously published multi-item scales with favorable psychometric properties. In addition to the review of pre-existing survey instruments from the literature, interviews were reviewed during the pilot study to identify context-specific details that warranted inclusion. The instrument was developed after a thorough review of several relevant studies on cyberbullying, Internet pornography use, Internet fraud, community bond, and PIU. The measurement items were modified to fit the adopted context of adolescent Internet usage behavior. This development followed the recommendations of MacKenzie et al. (2011) and the development procedures suggested by Devellis (2016) for standard psychometric scales. The measurement instrument for the questionnaire comprised 35 items measuring the four sections: (a) the cyberbullying, Internet pornography use, and Internet fraud scales; (b) the community bond scale; (c) the PIU scale; and (d) sociodemographic data questions. The first three sections used 4-point Likert scales (ranging from 1 strongly disagree to 4 strongly agree and 1 never to 4 always) to examine respondents’ perceptions of cyberbullying, Internet pornography use, and Internet fraud. The fourth section used a nominal scale to collect respondents’ basic sociodemographic information. The variables examined in the sociodemographic section included age, gender, high school grade, parental use of the Internet, parental restrictions on Internet use, parental involvement in adolescent Internet use, and parental evaluation of the Internet’s influence on the individual.

#### Cyberbullying Scale, Internet Pornography Scale, and Internet Fraud Scale

The cyberbullying construct was measured with three items adapted from Lazuras et al. (2017) and Savage and Tokunaga (2017). The Internet pornography construct was measured with five items adapted from several previous studies (Kor et al., 2014; Allen et al., 2017; Brown et al., 2017). The measurement items for the constructs of Internet fraud (seven items) was adapted from the measurements developed in various relevant studies (Vahdati and Yasini, 2015; Arora, 2016). Items were rated on 4-point Likert scales. In the pilot test, we collected data from 1,884 senior high school students in central Taiwan. The cyberbullying, Internet pornography, and Internet fraud constructs showed high reliability: 0.65 for cyberbullying, 0.92 for Internet pornography, and 0.87 for Internet fraud. The results showed that the coefficients for three constructs were higher than the minimum required value of 0.6 (Hair et al., 2010). Based on the reliability analysis results, the scales used in this study were satisfactory in terms of measuring the constructs of interest.

#### Community Bond Scale

The community bond measurement comprised seven items and was adapted from the studies of Teo et al. (2003), Buchan et al. (2006), Fiedler and Sarstedt (2014), and Tsai and Hung (2019). Items were rated on 4-point Likert scales. In the pilot test, the alpha coefficients for all items measured (α = 0.88) were higher than the minimum required value of 0.6 (Hair et al., 2010). Based on the reliability analysis results, the scales used in this study were satisfactory in terms of measuring the constructs of interest.

#### Problematic Internet Use Scale

The PIU measurement comprised six items and was adapted from the measurements developed by Shapira et al. (2000), Douglas et al. (2008), Lai and Kwan (2017), Wartberg et al. (2017), Faghani et al. (2020), and Musetti et al. (2020). Items were rated on 4-point Likert scales. The pilot test demonstrated high reliability with a Cronbach’s α of 0.84 and higher than the minimum required value of 0.6 (Hair et al., 2010). Based on the reliability analysis results, the scales used in this study were satisfactory in terms of measuring the constructs of interest.

### Sample and Descriptive Statistics

In the Taiwanese education system, children enter elementary school when they are 7 or 8 years old and complete this stage of their education by age 13 or 14. They then enter junior high school for 3 years followed by a further 3 years at senior high school. All three stages of education are compulsory. The years spent at junior high school are termed the 7th, 8th, and 9th grades, and those spent at senior high school (including vocational high school) are termed the 10th, 11th, and 12th grades.

This study was large scale and cross-sectional and used stratified single-stage cluster sampling. As part of the selection process, Taiwan was divided into regional blocks, and schools were randomly selected from each block. To avoid sampling bias toward any regional block, stratified sampling was performed using regional blocks as the strata. Based on the sampling frame, 60 senior high schools were selected, and three to four classes were randomly selected from each school. As a preliminary step, a teacher working in academic affairs at each high school was contacted to ensure their cooperation. This study adopted a quantitative survey and utilized mail and face-to-face interviews with high schools that were willing to distribute the survey. The teacher explained the questionnaire to the respondents. Data collection took ∼8 months. A total of 7,500 questionnaires were sent out simultaneously. All responses to the self-report instruments were collected during a regular school day in classrooms and in the presence of the class teacher. All participants of this study were students enrolled in the sample schools, and participation was voluntary.

A total of 7,034 questionnaires were returned, of which 1,823 were excluded because of excessive missing data, such as “don’t know” or “not applicable” answers, unspecified gender or grade, or inconsistent answers. Finally, a total of 5,211 respondents were included, and their data were analyzed. The response rate was 74.1%. Of the total 5,211 usable responses, 2,942 were from senior high school students, and 2,269 were from vocational high school students. The average age of the participants was 17.31 years [standard deviation (*SD*) = 0.95 years]; 54.5% were female and 45.5% were male. Regarding parental Internet use, 70.6% of the sample population indicated that their parents did use the Internet. Moreover, 53.6% indicated that their parents restricted their Internet use. Regarding parental involvement in adolescent Internet use, 54.7% indicated no involvement. For parents’ evaluation of the Internet’s influence on their children, 56.0% indicated both positive and negative effects. Table 1 shows the demographic and parent Internet usage characteristics of the sample.

**TABLE 1:** Profiles of respondents (*N* = 5,211).

Demographics/level	*N*	Percentage	Demographics/level	*N*	Percentage
Gender			Parental restrictions on Internet use		
Male	2,371	45.5	Yes	2,793	53.6
Female	2,840	54.5	No	2,418	46.4
Year in high school			Parental involvement in adolescent Internet use		
First	1,731	33.2	Yes	2,361	45.3
Second	2,001	38.4	No	2,850	54.7
Third	1,479	28.4	Parental evaluation of the Internet’s influence on you		
Parental use of the Internet			Positive influence	389	7.4
Yes	3,677	70.6	Both	2,917	56.0
No	1,534	29.4	Negative influence	749	14.4
			No influence	1,156	22.2

## Research Results

Structural equation modeling has the following benefits: it provides explicit modeling of measurement error, it estimates both direct and indirect relationships between latent variables, and it provides various indices of global model fit. The research hypotheses of this study were tested using partial least squares (PLS) regression with SPSS 18.0. PLS regression is component-based and employs a least squares estimation procedure. The psychometric properties of the variable measurement scales were also analyzed and missing data managed through list-wise deletion. This study used the measurement model to specify the relationships between the observed variables (manifest variables or indicators) and latent variables (constructs measured).

Depicting a model that contains moderators with PLS differs from traditional representations of the same research model. In a PLS model, the moderator (in this construct, treatment of personality traits) is an independent variable with a direct path to perceived benefit. Following the suggestion of Chin et al. (2003), these interaction measurement variables were calculated by multiplying every indicator in the moderator by every indicator in the independent variable. Conceptually, the interaction constructs (cyberbullying × community bond, Internet pornography use × community bond, Internet fraud × community bond) are depicted as having a direct path to perceived benefit. Additionally, this study used PLS to analyze the research model.

### Measurement Model Evaluation

Using PLS analysis, the composite reliability (CR) and average variance extracted volume (AVE) can assess the reliability and validity of the structural model, respectively. Accordingly, this study followed the recommendations of Bagozzi and Yi (2012) and selected the three most commonly used future evaluation indicators, which reflected the measurement mode. The three evaluation indicators were as follows: (1) item loadings (λ) and reliability coefficients (Cronbach’s alpha), (2) CR coefficients, and (3) AVE (Fornell and Larcker, 1981; Chin, 1998; Jöreskog and Sörbom, 2005; Hair et al., 2010; Bagozzi and Yi, 2012). Table 2 lists the indices of reliability and convergent validities for the scale.

**TABLE 2:** Validity and reliability.

Construct	Items	Mean	*SD*	Cronbach’s alpha	CR	AVE	DV
Cyber bullying	3	2.40	0.77	0.655	0.812	0.593	2.542
Internet pornography	5	1.42	0.69	0.922	0.942	0.765	4.235
Internet fraud	7	1.30	0.48	0.858	0.890	0.538	2.979
Community bond	7	2.65	0.84	0.925	0.939	0.689	1.451
Problematic Internet use (PIU)	6	2.05	0.78	0.871	0.903	0.608	1.281

**AVE, average variance extracted; CR, composite reliability; DV, discriminant validity. Discriminant validity = AVE/(correlation)^2^, where (correlation)^2^ = highest (correlation)^2^ between factors of interest and remaining factors*.*

The first indicator refers to the reliability of individual items (i.e., factor loadings) and is used to assess the factor loading of the latent variables and to test the statistical significance of each variable loading. In this study, the standardized item loadings ranged from 0.672 to 0.913, which indicated significance because all were higher than 0.60 (Hair et al., 2010). Internal consistency was assessed using the Cronbach’s alpha of each multi-item factor in the model. The Cronbach’s alpha ranged from 0.655 for cyberbullying to 0.925 for community bond, which suggested a high level of reliability. In addition, all constructs had a Cronbach’s alpha higher than the 0.60 benchmark.

The second indicator is the CR. The higher the CR, the higher the internal consistency reliability of the potential construct variable. Fornell and Larcker (1981) asserted that CR should be ≥0.6. The obtained CR coefficients ranged from 0.812 for cyberbullying to 0.942 for Internet pornography use, all higher than the 0.6 benchmark. Thus, the reliability of the scales in this study was confirmed.

The third indicator, AVE, calculates the explanatory power of the latent variables on the measured variables. Higher AVE values indicate that the potential variables have high discriminant and convergent validities. According to Fornell and Larcker (1981), the standard value of AVE for all constructs should exceed 0.5. In this study, the constructs possessed AVE values ranging from 0.538 for Internet fraud to 0.765 for Internet pornography use, all exceeding the threshold recommended by Fornell and Larcker (1981). Finally, the results of discriminant validity (DV) are shown in Table 2. The values of DV ranged from 1.281 for PIU to 4.235 for Internet pornography, and all constructs were >1.0, which supported the discriminant validity, thereby indicating an appropriate level of discriminate validity (Hair et al., 2017). Overall, the constructs thus demonstrated satisfactory reliability and validity. In summary, the internal reliability and validity results were acceptable, which enabled us to proceed to an estimation of the structural model.

### Testing the Moderating Effects

**FIGURE 2:**
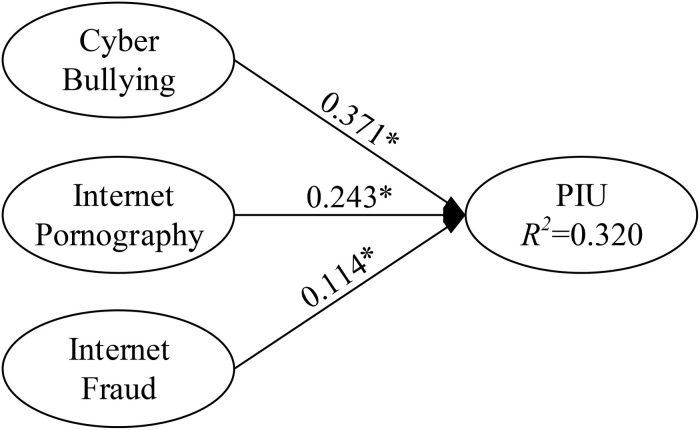
Path coefficients for the research model (excluding moderator main effect).

**FIGURE 3:**
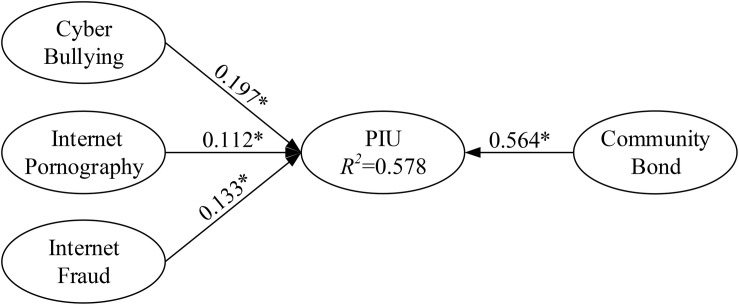
Path coefficients for the research model (including moderator main effect).

The purposes of the present study were to (1) determine the primary antecedents (cyberbullying, Internet pornography use, and Internet fraud) of PIU; (2) examine whether cyberbullying, Internet pornography use, Internet fraud, and community bond moderate or predict PIU; and (3) examine the effects of parental attitudes and behaviors toward the Internet on PIU. Regarding the moderating effects of community bond on adolescent PIU, this research used PLS regression to analyze and test the main effects of community bond. The moderating effects of community bond on PIU are illustrated in Figures 2, 3. For example, cyberbullying (predictor) and community bond (moderator) were multiplied to create an interaction construct (cyberbullying × community bond) for predicting PIU.

For PLS path modeling, this research followed the recommendations of Tenenhaus et al. (2005) in selecting goodness-of-fit to measure the structural model fitness. The researchers argued that the value should be ≥0.36; in the current study, the goodness-of-fit was 0.595, exceeding the baseline value. Thus, with an acceptable model fit, our measures were considered to be appropriate for the subsequent tests of the causal model and research hypotheses. Using PLS regression to estimate the path relationship between each research construct, the three hypothesized path relationships and the three hypothesized moderating effects, we discovered that four assumptions attained significance (*p* < 0.05).

**FIGURE 4:**
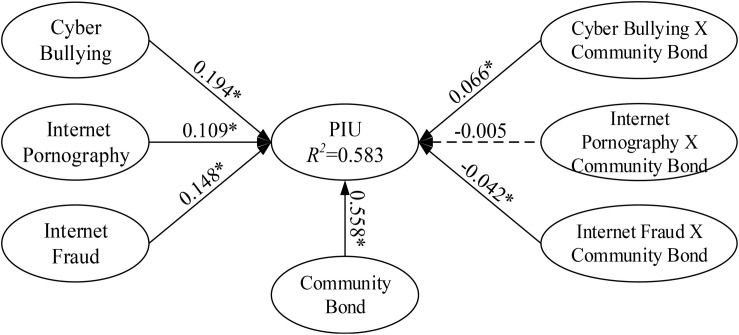
Path coefficients for the research model (including interaction effect).

To verify the analysis of the hypotheses and moderating effects, this study employed the moderator analysis method proposed by Baron and Kenny (1986). The moderating roles of community bond were determined according to the significance of the interaction terms in Model 3. Among the three hypothesized moderating effects, M2 were non-significant; specifically, community bond did not have moderating effects between Internet pornography use and PIU. Community bond positively moderated cyberbullying’s and Internet fraud’s effect on PIU (M1, beta = 0.066; M3, beta = −0.042, *p* < 0.05). As a result, community bond can be seen as the moderator of the effect of cyberbullying on PIU and Internet fraud on PIU. Figure 4 presents the full results of the moderation analysis, including the structural path estimates and explained variances.

The structural model path analysis results for the effect of Internet misconduct on PIU were as follows: cyberbullying, Internet pornography use, and Internet fraud were a significant determinant of PIU (β = 0.194, 0.109, and 0.148, respectively). The results related to predicting PIU were consistent with the cyberbullying, Internet pornography use, and Internet fraud hypotheses that were adapted to the context; thus, hypotheses 1–3 were supported. Cyberbullying, Internet pornography use, Internet fraud, and the interaction between cyberbullying and community bond explained 58.3% of the variance in PIU (*R*^2^ = 0.583). We also discovered that the proposed model explained a significant amount of variation in the endogenous variables. The endogenous variables exhibited strong explanatory powers of variation, which indicated the stability and robustness of the model. All estimated and standardized path coefficients (significant paths indicated with an asterisk) are indicated in Figure 4.

Table 3 presents the results of further investigation into the effects on PIU of cyberbullying, Internet pornography use and Internet fraud, and the interaction effect of community bond and cyberbullying (M1), Internet pornography (M2), and Internet fraud (M3) as moderated by parental use of the Internet, parental restrictions on Internet use, and parental evaluation of the Internet’s influence on the individual. First, participants whose parents did not use the Internet had the highest coefficients in all critical paths of PIU and the highest explanatory ability (*R*^2^ = 0.590) in their effects of cyberbullying on PIU. Second, participants whose parents placed restrictions on their Internet usage had the highest coefficients in all critical paths of PIU and the highest explanatory ability (*R*^2^ = 0.587) in their effects of cyberbullying on PIU. Third, participants whose parents evaluated their Internet usage as having a positive effect had the highest coefficients in all critical paths of PIU and the highest explanatory ability (*R*^2^ = 0.664) in their effects of Internet fraud on PIU (see Table 4).

**TABLE 3:** Estimation results for hypotheses.

Construct	Model 1		Model 2		Model 3	
	β	*t*-value	*B*	*t*-value	β	*t*-value
Cyber bullying → PIU	0.371*	29.828	0.197*	17.085	0.194*	17.754
Internet pornography → PIU	0.243*	16.377	0.112*	8.986	0.109*	8.009
Internet fraud → PIU	0.114*	9.325	0.133*	12.594	0.148*	12.506
**Moderator effect**						
Community bond → PIU			0.564*	56.309	0.558*	58.886
**Interaction effect**						
Cyber bullying × community bond → PIU					0.066*	6.182
Internet pornography × community bond → PIU					−0.005	0.366
Internet fraud × community bond → PIU					−0.042*	3.821
*R*^2^						
PIU	0.320		0.578		0.583	

*p < 0.05.

**TABLE 4:** Structural equation modeling (SEM) analysis results.

Construct	Parental use of the Internet		Parental restrictions on Internet use		Parental evaluation of the Internet’s influence on you			
			
	Yes (3,677)	No (1,534)	Yes (2,793)	No (2,418)	Positive influence (389)	Both (2,917)	Negative influence (749)	No influence (1,156)
Cyber bullying → PIU	0.189*	0.208*	0.222*	0.172*	0.144*	0.193*	0.259*	0.158*
Internet pornography → PIU	0.089*	0.161*	0.093*	0.132*	0.154*	0.109*	0.060*	0.134*
Internet fraud → PIU	0.167*	0.108*	0.133*	0.169*	0.344*	0.145*	0.089*	0.159*
**Moderator effect**								
Community bond → PIU	0.563*	0.545*	0.554*	0.551*	0.416*	0.558*	0.567*	0.548*
**Interaction effect**								
Cyber bullying × community bond → PIU	0.077*	0.039*	0.075*	0.056*	0.164*	0.060*	0.039	0.060*
Internet pornography × Community bond → PIU	–0.004	–0.008	0.003	–0.010	–0.084	–0.013	0.024	0.006
Internet fraud × Community bond → PIU	−0.051*	–0.024	−0.043*	−0.044*	–0.047	−0.033*	–0.048	–0.025
*R*^2^								
PIU	0.582	0.590	0.587	0.582	0.664	0.565	0.563	0.587

*p < 0.05.

## Discussion

Problematic Internet use can be defined as a condition in which an Internet user lacks the will to impose restrictions on their own online behavior despite recognizing the severe negative impacts of this behavior on their daily life (Tam and Walter, 2013; Spada, 2014). PIU has become a significant problem worldwide, especially among adolescents. To our knowledge, no previous empirical study has investigated the effects on PIU of inappropriate physical and mental health such as cyberbullying, Internet pornography use, and Internet fraud. The empirical results of the present study demonstrate that cyberbullying, Internet pornography use, and Internet fraud significantly and positively affect PIU, with cyberbullying exhibiting the strongest effect. Cyberbullying among adolescent Internet users is a recognized problem worldwide (AlBuhairan et al., 2017; Lazuras et al., 2017; Savage and Tokunaga, 2017). Social media outlets such as Facebook, Plurk, and Instagram have greatly expanded the number of online outlets that are available to students. The effects of these social media outlets on daily life has risen in line with their growing use for the discussion of classwork, sharing of information, holding of online activities, engagement in retail transactions, and search for friends with shared interests and backgrounds. Moreover, these effects have increased the opportunities for and the prevalence of phenomena such as cyberbullying, Internet pornography use, and Internet fraud.

The first instances of cyberbullying within an online community are frequently overlooked or downplayed as teasing among peers. Self-awareness of cyberbullying within a community often comes only after the phenomenon has already increased significantly in scope and severity. Long-term exposure to bullying is known to affect the mentality, daily life, and PIU-related behaviors of students. In terms of Internet pornography, easy and free access to online pornographic material render this medium particularly harmful to students’ physical and mental health. Moreover, the difficulties faced in self-regulating pornography use have helped make online pornography the most popular form of pornography currently accessed by students. The anonymity and privacy of general online activity provide Internet users with significant protection from outside scrutiny, allowing them to reveal and share their innermost erotic tendencies online, making pornography one of the most searched for subjects on the Internet and causing students to engage in problematic Internet or Internet pornography use. Finally, in terms of Internet fraud, online shopping and auction websites have popularized e-commerce; countless online retailers as well as adolescent entrepreneurs now market and sell products through social media at significantly discounted prices. However, students’ online purchases are frequently affected by types of fraud, such as products that fall short of buyer expectations, products that are not delivered as promised, products with missing components, and products that are otherwise defective. Therefore, Internet fraud is another behavior that affects PIU. In school settings, teacher supervision largely keeps student participation in cyberbullying, Internet pornography, and Internet fraud under control. After school, however, students revert to their usual patterns of online behavior. Thus, counselors and teachers should place greater emphasis on developing effective strategies to prevent these inappropriate online behaviors. One suggestion is that students should seek assistance from their teachers and parents immediately upon encountering these behaviors. Moreover, school counselors and teachers should pay particular attention to the physical and mental health of students that have already received related counseling to prevent these students from re-engaging in these behaviors.

When participating in the online community and helps deliver emotional and practical value to individual members. The interaction effects between cyberbullying and community bond and between Internet fraud and community bond have a statistically significant effect on PIU. Moreover, community bond was found to have a significant and positive effect on PIU, indicating a positive correlation between the degree of association of students with members in an online community and consistency in its activities. Thus, participation in an online community that engages in harmful or negatively aligned activities is expected to increase PIU. However, the interaction effect between cyberbullying and community bonds shared by a student may moderate this effect on PIU. Moreover, it has been consistently shown that students with more community bonds use the distinctive characteristics of their online communities to validate the characteristics or values that they share with members of these communities. An adolescent, who upon becoming aware of a bullying incident in their online community joins in or otherwise validates this behavior out of a desire to gain the approval of this community, would be expected to have a higher level of PIU. Therefore, the findings of the present study suggest that parents and teachers pay closer attention to the behaviors and activities of students on the Internet at home and at school and that these adults work proactively to reduce the potential negative effects of students’ online activities. Bullying, infrequently a short-term phenomenon, tends to have cyclical repercussions when it takes place online. The victim of a cyberbullying attack often has a desire to respond, which may encourage others to join the fray leading to a potentially long and drawn-out series of increasingly abusive and antagonistic communications. In this situation, parents and teachers can play a positive role in informing students about the mindset and attitudes necessary to appropriately handle the situation. In addition, assistance from school administration may be sought to help further reduce the effect of cyberbullying.

The interaction between Internet fraud and community bond has a significant negative effect on PIU. As a relatively new form of crime, Internet fraud can occur in multiple forms, and it affects the daily lives of numerous people (Vahdati and Yasini, 2015; Arora, 2016; Moreno-Fernández et al., 2017). Thus, when students recognize Internet fraud as a significant problem, it may affect PIU. However, the interaction effect between the community bond of a student and Internet fraud may reduce this effect on PIU. When adolescents become aware of the severity of Internet fraud, they will search for information about new Internet fraud methods shared by members in an online community. From this information, they learn about new Internet fraud methods; thus, the effect on PIU decreases. Therefore, the results of this study suggest that, although students become aware of new Internet fraud methods on online communities, school administration should also pay close attention to stay updated on Internet frauds and communicate these to students to prevent them from being scammed.

Previous studies have pointed out that parental relationships, parent–child relationships, and parents’ Internet behavior are related to adolescents’ Internet use (Pontes et al., 2016; Lai and Kwan, 2017; Wartberg et al., 2017; Musetti et al., 2020; Sela et al., 2020). The results showed that with regard to the two variables “parental use of the Internet” and “parental restrictions on Internet use,” having parents who were unable to use the Internet and having parents who placed time-based restrictions on Internet use most significantly altered the effect of cyberbullying on PIU. Despite the Internet’s prevalence and the fact that most students are introduced to the Internet at an early age, a significantly small percentage of the students’ parents was unable to use the Internet effectively. Survey results indicated that nearly 30% of the parents did not know how to use the Internet. Such lack of knowledge severely undermines the ability of parents to understand the online activities of their children and prevents them from assisting their children to resolve problems encountered online. Parents who lack Internet knowledge are thus unable to provide appropriate guidance when their child encounters cyberbullying, which increases the severity of the effect of bullying on PIU. Regarding the variable “parental evaluation of the Internet’s influence on the participant,” the effect of cyberbullying on PIU was identified as relatively weak when parents perceived the Internet as having an overall positive effect on their child for reasons that included parental awareness of the importance of the Internet in everyday life, the practical benefits of Internet use such as information searches and online learning, and parent–child discussions already held regarding proper Internet use and online behavior. Additionally, PIU is a family-related problem rather than a problem that adolescents should handle alone. Parents must be sufficiently cognizant of and sensitive to their own Internet and Internet-related behaviors to avoid sending non-verbal signals to their children that suggest approval of inappropriate behaviors and to better prevent their children from engaging in PIU.

## Conclusion

The research results of this study demonstrated that cyberbullying, Internet pornography use, Internet fraud, and community bond significantly and positively affect PIU in adolescents. Additionally, community bond has a significant moderating effect on the relationship between cyberbullying, Internet fraud, and PIU. This study revealed that PIU and inappropriate physical and mental health have become serious problems among adolescents and that the planning and implementation of preventive and control measures are urgently required in Taiwan. To our knowledge, this study is the first to investigate the relationship between parental Internet attitude and behaviors and adolescent Internet usage behavior. Because of the lack of similar studies, the results of this study can be considered unique; parental use of the Internet, parental restrictions on Internet use, and parental involvement in adolescent Internet use have significant effects on PIU.

## Limitations and Future Research

This study had some limitations. First, other factors that may influence PIU such as average time spent online and adolescent age were not considered. These and other relevant factors may be explored using the same model in the future. Second, relevant studies have relied on participants self-reporting their inappropriate physical and mental health such as cyberbullying, Internet pornography use, and Internet fraud, which may elicit misreporting to avoid judgment and cause common method variance. Although the present study also used a self-report inventory, the items asked participants to measure the target behaviors of other adolescents based on the respondent’s actual online experiences. Third, the present research was conducted at a time when most Internet users in Taiwan were using 3G mobile telecommunications technology. Thus, the results may not be linearly extrapolated to describe student PIU in today’s age of “anytime, anywhere” 4G mobile Internet. Future research may use the present research model to study adolescents in the 4G environment to describe the current effects on PIU of the three types of physical and mental behaviors; this would improve the comprehensiveness and representative nature of the model and promote the linear extrapolation of the results across mobile telecommunications technology platforms. Finally, because the present study only collected data at one point in time, no inferences were possible regarding the relationship between changes in parental Internet attitudes and behaviors and changes in the time that students spend online or the changes in PIU over time; therefore, a longitudinal study is recommended to observe these relationships.

## Data Availability Statement

The raw data supporting the conclusions of this article will be made available by the authors, without undue reservation, to any qualified researcher.

## Ethics Statement

Ethical review and approval was not required for the study on human participants in accordance with the local legislation and institutional requirements. Written informed consent to participate in this study was provided by the participants’ legal guardian/next of kin.

## Author Contributions

C-MC: data collection, concept and design, statistical analysis, interpretation of data, and writing up. K-YK: data collection, interpretation of data, and writing up. T-KY: obtaining funding, data collection, statistical analysis, interpretation of data, and study supervision. All authors wrote the manuscript together and approved the final manuscript.

## Conflict of Interest

The authors declare that the research was conducted in the absence of any commercial or financial relationships that could be construed as a potential conflict of interest.
