# Supplementary material for: Reactions to Problematic Internet Use Among Adolescents: Inappropriate Physical and Mental Health Perspectives
Source: Front Psychol. 2020 Jul 31;11:1782. doi: 10.3389/fpsyg.2020.01782 (PMC7411128; doi:10.3389/fpsyg.2020.01782)
Supplement: Supplementary file 1 [file Table_1.DOCX]

**Appendix A: Research questionnaire**

|  |  | *Strongly Disagree* | *Disagree* | *Agree* | *Strongly Agree* |
| --- | --- | --- | --- | --- | --- |
| 1 | If I were bullied, it is safer to express my resentment online. | □ | □ | □ | □ |
| 2 | If I were attacked/mocked online, it is fine to fight back anonymously. | □ | □ | □ | □ |
| 3 | If I saw hateful content against others online, I would tell my teacher. | □ | □ | □ | □ |
| 4 | It fuels an interest I have to understand more about sex. | □ | □ | □ | □ |
| 5 | It gives me a sense of excitement. | □ | □ | □ | □ |
| 6 | When I use it to feel physical pleasure | □ | □ | □ | □ |
| 7 | When I use it to expand my knowledge about sexual possibilities. | □ | □ | □ | □ |
| 8 | When I use it to avoid feeling uncomfortable or unpleasant emotions. | □ | □ | □ | □ |
| 9 | I find it dangerous to shop on the Internet. | □ | □ | □ | □ |
| 10 | I am concerned that I may not get what I ordered online. | □ | □ | □ | □ |
| 11 | I feel shopping on the Internet entails uncertainty or vulnerability. | □ | □ | □ | □ |
| 12 | I believe Internet fraud is a significant problem. | □ | □ | □ | □ |
| 13 | Society recognizes Internet fraud as a significant societal problem | □ | □ | □ | □ |
| 14 | My friends recognizes Internet fraud as a significant societal problem. | □ | □ | □ | □ |
| 15 | Additional legislation is needed to deter Internet fraud. | □ | □ | □ | □ |
| 16 | I am a valuable member of the community group. | □ | □ | □ | □ |
| 17 | I am an important member of the community group. | □ | □ | □ | □ |
| 18 | I am proud to tell others that I am part of this community group. | □ | □ | □ | □ |
| 19 | Other community group members and I share the same objectives. | □ | □ | □ | □ |
| 20 | The friendships I have with other community group members mean a lot to me. | □ | □ | □ | □ |
| 21 | If the community group members planned do something, I’d think of it as something ‘we’ would do. | □ | □ | □ | □ |

|  |  | *Never* | *Seldom* | *Usually* | *Always* |
| --- | --- | --- | --- | --- | --- |
| 22 | Do you feel depressed, moody, or nervous when you are off-line, which goes away once you are back online? | □ | □ | □ | □ |
| 23 | Do you feel restless, moody, depressed, or irritable when attempting to cut down or stop Internet use? | □ | □ | □ | □ |
| 24 | Do you feel the need to use the Internet with increased amounts of time in order to achieve satisfaction? | □ | □ | □ | □ |
| 25 | Do you find that you stay online longer than you intended? | □ | □ | □ | □ |
| 26 | Do you find yourself anticipating when you will go online again? | □ | □ | □ | □ |
| 27 | Do you try to cut down the amount of time you spend online and fail? | □ | □ | □ | □ |
| 28 | Do others in your life complain to you about the amount of time you spend online? | □ | □ | □ | □ |

**Respondents’ Basic Information**

1. What is your gender? □Male □Female

2. How old are you? year

3. What is your grade? □1^st^ Grade □2^nd^ Grade □3^rd^ Grade

4. Parental use of the Internet? □Yes □No

5. Parental restrictions on Internet use? □Yes □No

6. Parental involvement in adolescent internet use? □Yes □No

7. Parental evaluation of the Internet's influence on you?

□Positive Influence □Both □Negative Influence □No Influence
